# Prevalence and Risk Factors of Diabetes and Diabetic Retinopathy in Liaoning Province, China: A Population-Based Cross-Sectional Study

**DOI:** 10.1371/journal.pone.0121477

**Published:** 2015-03-18

**Authors:** Yuedong Hu, Weiping Teng, Limin Liu, Kang Chen, Lei Liu, Rui Hua, Jun Chen, Yun Zhou, Lei Chen

**Affiliations:** 1 Department of Ophthalmology, the First Affiliated Hospital of China Medical University, Shenyang City, Liaoning Province, the People’s Republic of China; 2 Diabetic Eye Center of Liaoning province, Shenyang City, Liaoning Province, the People’s Republic of China; 3 Department of Endocrinology, the First Affiliated Hospital of China Medical University, Shenyang City, Liaoning Province, the People’s Republic of China; 4 The key Laboratory of Endocrine diseases of Liaoning Province, Shenyang City, Liaoning Province, the People’s Republic of China; Saitama Medical University, JAPAN

## Abstract

**Aim:**

To evaluate the prevalence and risk factors of diabetes and diabetic retinopathy (DR) in northeast area of China with a population-based study.

**Methods:**

A population of 3173 (aged from 20 to 80 years old) was stratified by geographical location and age in Liaoning province, China. Prediabetes and diabetes were diagnosed according to the guideline of American Diabetes Association. Retinal photographs were obtained by using digital non-mydriatic camera for the presence and grading of DR according to the modified ETDRS Airlie house classification. Blood samples and comprehensive questionnaires were obtained for evaluation of laboratory results and risk factors.

**Results:**

The prevalence of prediabetes and diabetes was 20.7% and 10.4%, respectively. Among diabetes patients, DR prevalence was 11.9%. Age, obesity, total cholesterol, triglycerides, hypertension, living in rural areas and diabetes family history are all risk factors for prediabetes and diabetes. Waist-to-hip circumference rate served as a better obesity index to estimate diabetes risk compared with body mass index and waist circumference. Among all risk factors that we investigated, only the length of diabetes history was associated with the incidence of DR. However, DR prevalence in the newly discovered patients in rural areas was significantly higher than that in urban areas.

**Conclusion:**

According to this study, 1 in 10 people has diabetes, 2 in 10 people have prediabetes, and 1 in 10 diabetics has DR in Liaoning province. In rural areas, diabetes was poorly recognized with limited medical resources, which probably resulted in more diabetes patient at a high risk of DR.

## Introduction

The prevalence of diabetes has increased worldwide, especially in the Asia Pacific region and China [1, 2]. According to the national survey of China National Diabetes and Metabolic Disorder Study Group, the age-standardized prevalence of diabetes in China rose to 9.7% between 2007 and 2008, which almost doubled in the past decade and affected 92.4 million adults (age ≥ 20 years) [2]. It is estimated that there will be 380 million people with type 2 diabetes and 418 million people with impaired glucose tolerance (IGT) by the year of 2025 worldwide [3]. Though numerous studies have been conducted to analyze risk factors and predictors of diabetes for the disease control, the undergoing increase of diabetes incidence still requires a better understanding of risk factors.

Diabetic retinopathy, which is a common complication in diabetes, is characterized by retinal vascular leakage, inflammation and abnormal neovascularization [4, 5]. DR is recognized as a leading cause of blindness and visual impairment in working-age adults in developed and developing countries [6, 7]. The World Health Organization (WHO) lists DR as a priority disease in their “VISION 2020” program initiative for the global elimination of avoidable blindness. Potential risk factors of DR include age, duration of diabetes, glycemic level, blood pressure, pregnancy and nephropathy [8–11]. However, risk reduction for DR with glucose and BP control in diabetic patients is limited [12, 13]. The prevalence and risk factors of DR in Chinese have been reported by several studies, but varied because of the research design, sampling size and complexities [1, 8, 11, 14]. In addition, most studies on DR were hospital based, leading to estimation bias on the incidence and risk factors of DR in general population. To date, there is rare population-based study of the prevalence of DR in mainland China. Thus, in the present study, we conducted a population-based study in Northeast China to provide an estimate on prevalence of diabetes and DR in this region and a better understanding of related risk factors.

## Materials and Methods

### Study subjects

**Fig 1:**
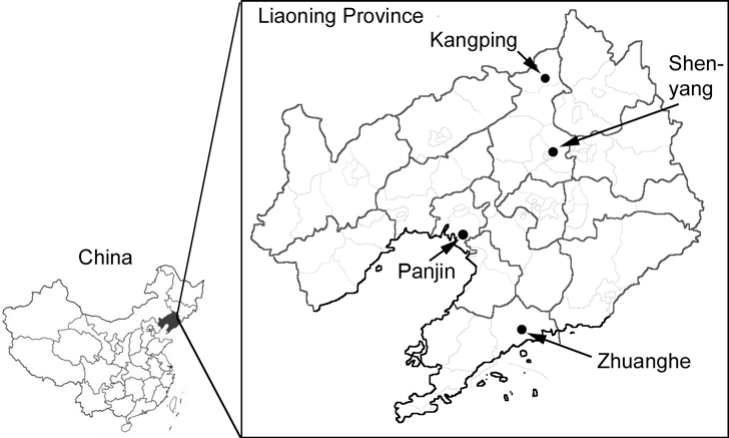
Map of study areas in Liaoning Province, China. The study subjects were stratified according to the geographic region (north, east, and south of Liaoning province) and the degree of civilian (urban and rural). Subjects from urban were randomly sampled from five communities in the five administrative districts of Shenyang city. Subjects from rural were randomly selected from 2 natural villages located in east Panjin, 2 natural villages located in south Zhuanghe and 2 natural villages located in north Kangping.

A multistage, stratified sampling method was used to select representative study subjects aged from 20 to 85 in the general population of Liaoning province, China. The sampling was stratified according to the geographic region (north, east, and south of Liaoning province) and the degree of civilian (urban and rural) (Fig. 1). In urban areas, subjects were randomly sampled from five communities in the five administrative districts of Shenyang city. In rural areas, subjects were randomly selected from 2 natural villages located in east Panjin, 2 natural villages located in south Zhuanghe and 2 natural villages located in north Kangping (Fig. 1). The subjects were stratified according to the age distribution of Chinese population based on 2006 national census. Only people who had lived in their current residence for no less than 5 years were eligible for participation. Totally 3345 people were admitted to the study, and 3173 people (1,032 men and 2141 women) completed the study. The response rate was 94.8%. The ratio of urban to rural participants in the present study was 51.9 to 48.1, whereas the ratio of urban to rural general population of Liaoning province in 2006 was 58.99 to 41.01. We collected study subjects according to the degree of civilian of Liaoning province. However, part of subjects from Shenyang area were living in a rural life style (working on the farm, low income, living far from downtown, and etc.) though their official residence (named Hukou in China) was identified as urban.

Research protocols were approved by the Ethics Committee of China Medical University. We have complied with the Declaration of Helsinki Ethical Principles for medical research involving human subjects. Written informed consent was obtained from each participant before data collection.

### Health examination survey

Information on demographic characteristics, socioeconomic status, educational level, occupation, income, personal and family medical history, and probable risk factors for diabetes and DR was obtained by trained staff with a standard questionnaire. Cigarette smoking was defined as having smoked at least 100 cigarettes in the past. Alcohol consumption was defined as having at least 30 g of alcohol per week for at least 1 year. Regular exercise was defined as participation in at least 30 minutes of moderate-intensity aerobic activity, no less than 3 days per week.

Blood pressure (BP) was measured on the right arm in the sitting position using a standard mercury sphygmomanometer after at least 5 minutes of rest. The first and fifth Korotkoff sounds were recorded. Body weight, height and circumference of waist and hip were measured with standard methods as described previously [15]. Body weight was measured with electronic scales to the nearest 0.1 kg. Body height was measured to the nearest 0.1 cm by using a stadiometer. Girth measurements, recorded to the nearest 0.1 cm, were taken with a cloth tape. Waist circumference (WC) was measured at the midline between the lower border of the ribs and the iliac crest (usually at a level of 2.5 cm above the umbilicus) in the horizontal plane after a normal expiration. Hip circumference was defined as the maximum girth reading between waist and thigh. Two measurements were taken, with tolerances of < 5 mmHg for blood pressure, < 1 kg for weight, and < 1 cm for height and circumference. A third measurement was taken if the difference between the first two exceeded the tolerance. The mean of the replicates was used in the analysis. Body mass index (BMI) was calculated as weight in kilograms divided by height in meters squared (kg/m^2^). Waist-to-hip circumference rate (WHR) was calculated using the direct measurements. All study investigators and staff members completed a training program that familiarized them with the aim of the study and the specific tools and methods used.

### Laboratory assays

Participants were instructed to maintain their usual physical activity and diet for at least 3 days before the oral glucose-tolerance test (OGTT). After at least 10 hours of overnight fasting, a venous blood specimen was collected into a vacuum tube containing sodium fluoride. Participants with no history of diabetes were given a standard 75 g of glucose saluted in water, whereas for safety sake, participants with a self-reported history of diabetes were given a steamed bun that contained approximately 80 g of complex carbohydrates. The diagnosis of diabetes depended on the result of the modified OGTT. Blood samples were drawn at 0, 30, and 120 minutes after the glucose or carbohydrate load to measure plasma glucose and insulin concentrations. Plasma glucose concentrations were measured by Hexokinase end point method. Plasma insulin concentration was measured by chemiluminescent analysis. Homeostasis model insulin resistance index (HOMA-IR) = fasting glucose (mmol/L) x fasting insulin (FINS) concentration (mIU/L) /22.5. Serum total cholesterol (TC), triglycerides (TG) and high-density lipoprotein cholesterol (HDL-C) levels were measured using an automatic biochemical analyzer (AU1000, Olympus, Japan). Plasma TSH, FT_3_ and FT_4_ were measured by solid-phase chemiluminescence immunoassay (IMMULITE1000, DPC, USA).

Diabetes and prediabetes were diagnosed according to the criteria of American Diabetes Association [16]. Diabetes was defined as fasting plasma glucose (FPG) ≥ 7.0mmol/l or 2-hr value in (2-hr OGTT) ≥ 11.1mmol/l (200 mg/dl) or self-reported diabetes medication use. Individuals with impaired fasting glucose (IFG) (FPG ≥ 5.6mmol/l and < 6.9 mmol/L) and/or impaired glucose tolerance (IGT) (2-hr OGTT of 7.8 mmol/L to 11.0 mmol/L) without medication were defined as having prediabetes.

### Ophthalmological examination

Retinal photographs were obtained using the non-mydriatic digital fundus camera (TRC-NW6S/7S, TOPCON, Japan) after visual acuity and slit lamp microscope examination. For each participant, one image for each eye centered on the fovea (45°) was taken in the physiologically dilated pupil after dark adaptation [17]. Each image was graded in a masked manner by two trained oculist separately for the presence of retinopathy lesions, following the modified Early Treatment Diabetic Retinopathy Study (ETDRS) [18]. If the grades were different, the other oculist who was trained in ophthalmology department of the University of Hong Kong would give the final diagnosis. The level of retinopathy for each eye was determined and the individual classification was based upon the worse eye. There were 35 patients that could not get a clear fundus image for anterior segment refractive media opacity. They accepted mydriasis and binocular indirect ophthalmoscope by one of the two oculists who reviewed retinal images.

### Statistical analysis

Statistical analyses were conducted using the SPSS software (version 13.0, SPSS, Chicago, IL, USA). Age- and/or gender-adjusted comparisons of characteristics by prediabetes and diabetes or DR were conducted with analysis of covariance (ANOVA) tests. Receiver operating characteristic (ROC) curve was generated to obtain the values of area under the curve (AUC) for each obesity index (WHR, WC and BMI) as a predictor of diabetes. Logistic regression was used for analysis of risk factors for prediabetes, diabetes and DR. All analyses were two-tailed with *p* < 0.05 as statistical significance.

## Results

Finally, a total of 3173 subjects were eligible for participation in the present study. Among these 3173 participants, 329 (10.4%) were diagnosed with diabetes (aged from 22 to 84) according to their disease history, FPG or OGTT, and 657 (20.7%) were diagnosed with prediabetes (aged from 20 to 78). Incidence of any form of DR in the diabetes patients was 11.9% including 31 subjects with non-proliferative DR and 8 subjects with proliferative DR. The presence of isolated retinopathy signs (e. g. retinal haemorrhages, microaneurysms, hard exudates and cotton wool spots) is known to occur in older people without diabetes, named as “DR-like retinopathy” in this paper. The prevalence of DR-like retinopathy was 1.4% and 0.3% in prediabeteic and non-diabetic subjects, respectively.

Age-and/or gender- adjusted characteristics of non-diabetic, prediabetic and diabetic populations were presented in Table 1. In the age- and gender-adjusted comparison, there was no difference in alcohol consumption, regular exercise habit or plasma TSH levels between study subjects according to the presence of prediabetes or diabetes. Adjusted age, gender and family diabetes history were significantly different between groups. Populations with prediabetes or diabetes were older than non-diabetic control. There were more males in the diabetic population than in prediabetic or non-diabetic populations. The percentage of people with diabetes family history was significantly higher in prediabetes and diabetes populations compared with non-diabetic control. Levels of FPG, OGGT, FINS, HOMA-IR, TG and TC were significantly higher in people with diabetes than those without diabetes. Systolic/diastolic blood pressure, BMI and WHR were higher in people with prediabetes or diabetes than control. INS 30 significantly decreased in people with diabetes than those without diabetes. INS 120 significantly increased in people with prediabetes or diabetes compared with control, together with FPG and OGGT, indicating insulin resistance in people with prediabetes or diabetes.

**Table 1:** Weighted population and clinical characteristics of healthy, prediabetes and diabetes subjects.

	Healthy	Prediabetes	Diabetes	*p*
N	2187 (68.9%)	657 (20.7%)	329 (10.4%)	
Age (years)	39.4^a^ (38.7, 40.1)	45.6^b^ (44.3, 46.9)	53.2^c^ (51.3, 55.1)	< 0.001
Male (%)	34.8^a^ (32.8, 36.9)	35.0^a^ (31.2, 38.8)	48.1^b^ (41.8, 54.4)	< 0.001
Smoking (%)	23.1^a^ (21.6, 24.6)	18.2^b^ (15.5, 21.0)	20.3^ab^ (16.2, 24.5)	0.002
Alcohol consuming (%)	18.7 (17.3, 20.1)	16.0 (13.4, 18.6)	16.6 (12.8, 20.5)	> 0.05
Regular exercise (%)	33.2 (31.2, 35.1)	30.7 (27.1, 34.3)	32.9 (27.6, 38.2)	> 0.05
DM in the family (%)	14.9^a^ (13.3, 16.5)	21.6^b^ (18.6, 24.6)	29.9^c^ (25.4, 34.3)	< 0.001
FPG (mmol/L)	5.02^a^ (4.97, 5.07)	5.58^b^ (5.49, 5.67)	8.62^c^ (8.48, 8.75)	< 0.001
OGGT (mmol//L)	5.86^a^ (5.78, 5.94)	8.57^b^ (8.42, 8.73)	15.75^c^ (15.52, 15.98)	< 0.001
FINS (mIU/L)	10.0^a^ (9.23, 10.8)	10.8^a^ (9.37, 12.4)	16.0^b^ (13.7, 18.2)	< 0.001
INS30 (mIU/L)	59.1^a^ (56.8, 61.3)	55.6^a^ (51.5, 59.8)	35.1^b^ (29.0, 41.3)	< 0.001
INS120 (mIU/L)	40.5^a^ (38.3, 42.7)	70.4^b^ (66.3, 74.5)	56.7^c^ (50.6, 62.8)	< 0.001
Systolic blood pressure (mmHg)	124.3^a^ (123.5, 125.2)	130.2^b^ (128.8, 131.7)	131.6^b^ (129.4, 133.7)	< 0.001
Diastolic blood pressure (mmHg)	79.5^a^ (79.0, 80.0)	83.7^b^ (82.9, 84.6)	83.3^b^ (82.0, 84.6)	< 0.001
BMI (kg/m^2^)	23.9^a^ (23.7, 24.0)	25.5^b^ (25.2, 25.8)	25.3^b^ (24.9, 25.7)	< 0.001
WHR	0.841^a^ (0.838, 0.844)	0.873^b^ (0.868, 0.879)	0.881^b^ (0.872, 0.889)	< 0.001
HOMA-IR	2.24^a^ (1.89, 2.60)	2.71^a^ (2.05, 3.37)	6.77^b^ (5.80, 7.74)	< 0.001
TG (mmol/L)	1.46^a^ (1.40, 1.52)	1.87^b^ (1.76,1.98)	2.43^c^ (2.27, 2.59)	< 0.001
TC (mmol/L)	4.66^a^ (4.62, 4.71)	4.91^b^ (4.83, 4.99)	5.10^c^ (4.98, 5.22)	< 0.01
HDL-C (mmol/L)	1.41^a^ (1.40, 1.43)	1.40^ab^ (1.38, 1.43)	1.37^b^ (1.33, 1.41)	0.034
TSH (mIU/L)	2.28 (2.14, 2.42)	2.49 (2.13, 2.85)	2.66 (2.24, 3.07)	> 0.05

Age was adjusted by gender. Gender was adjusted by age. Other variables (FPG, OGGT120, FINS, INS30, INS120, systolic blood pressure, diastolic blood pressure, GMI, WHR, HOMA-IR, TG, TC, HDL-C and TSH) were adjusted by age and gender. Different superscripts indicate significantly statistical difference between groups (*p* < 0.05).

**Fig 2:**
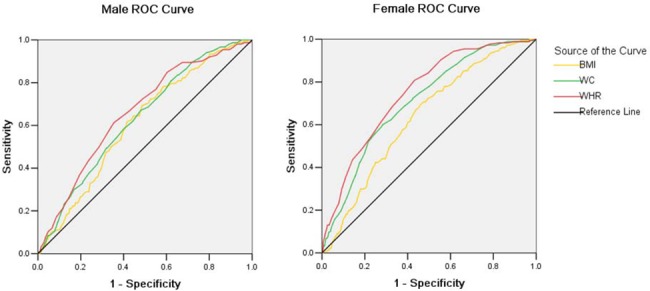
Receiver operating characteristics curve (ROC). BMI: body mass index; WC: waist circumference; WHR: waist hip ratio.

To compare different obesity indices for diabetes, ROC curves were applied. Fig. 2 showed gender-specific areas under ROC curves with corresponding validity parameters of different obesity indices in predicting diabetes. For both male and female, WHR yielded the highest AUC (0.659 and 0.718, respectively) followed by WC and BMI, indicating WHR is a better index used for diabetes risk assessment, at least in our study population.

Age and/or gender-adjusted characteristics of populations with diabetes by the presence of DR were shown in Table 2. There was no difference in the presence of DR by age or gender. In the age- and gender-adjusted comparison, there was no difference in smoking, alcohol consumption, regular exercise, family diabetes history, FPG, INS30/120, systolic/diastolic blood pressure, WHR, TG, HDL-C or TSH. People with DR had longer diabetes history and higher OGGT, FINS and HOMA-IR, but lower BMI and TC compared with those without DR.

**Table 2:** Weighted clinical characteristics of diabetes population by the presence of DR.

	NDR	DR	*p*
N	290	39	
Age (years)	54.4 (53.1, 55.7)	56.8 (53.4, 60.3)	0.196
Men (%)	45.4 (39.6, 51.1)	48.8 (32.7, 64.9)	0.692
smoking (%)	25.9 (21.3, 30.4)	17.4 (4.90, 29.8)	0.210
Alcohol consuming (%)	20.2 (16.1, 24.2)	18.0 (6.81, 29.1)	0.720
Regular exercise (%)	39.5 (33.9, 45.1)	33.4 (18.0, 48.7)	0.462
Family diabetes history (%)	28.6 (23.3, 33.9)	31.9 (17.4, 46.4)	0.667
Diabetes history (year)	1.99^a^ (1.50, 2.47)	5.00^b^ (3.71, 6.29)	< 0.001
FPG (mmol/L)	8.50 (8.13, 8.87)	9.55 (8.54, 10.6)	0.056
OGGT (mmol//L)	15.6^a^ (15.1, 16.2)	18.0^b^ (16.4, 19.6)	0.008
FINS (mIU/L)	13.1^a^ (9.66, 16.6)	24.8^b^ (15.3, 34.2)	0.024
INS30 (mIU/L)	31.9 (27.1, 36.7)	35.6 (22.3, 48.8)	0.613
INS120 (mIU/L)	55.7 (48.4, 63.0)	50.7 (31.0, 70.5)	0.643
Systolic blood pressure (mmHg)	138.8 (136.3, 141.4)	135.8 (128.9, 142.6)	0.412
Diastolic blood pressure (mmHg)	85.1 (83.7, 86.5)	83.4 (79.6, 87.2)	0.392
BMI (kg/m^2^)	25.7^a^ (25.4, 26.1)	24.5^b^ (23.5, 25.4)	0.017
WHR	0.899 (0.891, 0.906)	0.894 (0.873, 0.914)	0.644
HOMA-IR	4.83^a^ (2.14, 7.52)	14.94^b^ (7.71, 22.2)	0.011
TG (mmol/L)	2.56 (2.25, 2.86)	1.6^b^ (0.856,2.52)	0.055
TC (mmol/L)	5.40^a^ (5.23, 5.57)	4.80^b^ (4.34, 5.26)	0.017
HDL-C (mmol/L)	1.38 (1.34, 1.43)	1.36 (1.24, 1.49)	0.797
TSH (mIU/L)	2.79 (1.98, 3.59)	1.71 (0.608, 4.03)	0.389

Age was adjusted by gender. Gender was adjusted by age. Other continuous variables were adjusted by age and gender. Different superscripts indicate significantly statistical difference between groups (*p* < 0.05).

Table 3 showed the results of logistic regression analysis for prediabetes and diabetes with risk factors (age, gender, smoking, alcohol consumption, regular exercise, residential region, diabetes family history, hypertension, WHR, TC, TG and HDL-C). Age, WHR, TC, TG, hypertension, living in rural areas and diabetes family history are risk factors for both prediabetes and diabetes. Surprisingly, smoking appeared to be a protective factor of prediabetes. Binary logistic regression was applied to analyze risk factors (age, gender, smoking, alcohol consumption, BMI, WHR, hypertension, TC, TG, HDL-C, residential region, HOMA-IR and years of diabetes history) for DR in diabetes patients. Only duration of diabetes history was significantly associated with the incidence of DR (OR = 1.11, *p* = 0.011). Moreover, 44.8% diabetes patients (65 subjects) were newly diagnosed in urban areas, whereas 70.6% diabetes patients (130 subjects) were newly diagnosed in rural areas. The proportion of new diabetes patients was significantly different between urban and rural areas (*p* < 0.001). DR incidence in these new diabetes patients was 4.6% (3 subjects) in urban areas and 10.0% (13 subjects) in rural areas, respectively (urban vs rural, *p* = 0.040).

**Table 3:** Multivariate logistic regression analysis of risk factors of diabetes.

	Prediabetes		Diabetes	
Age	OR (95% CI)	*p*	OR (95% CI)	*p*
40–65 vs 18–40	1.69 (1.35, 2.12)	<0.001	4.07 (2.74, 6.04)	< 0.001
65- vs 18–40	2.08 (1.52, 2.83)	<0.001	7.13 (4.52, 11.2)	< 0.001
Male	0.95 (0.74, 1.22)	0.662	1.40 (1.01, 1.94)	0.044
Smoking	0.70 (0.54, 0.92)	0.010	0.79 (0.57, 1.11)	0.178
Alcohol consumption	0.82 (0.60, 1.10)	0.186	0.72 (0.49, 1.04)	0.082
Regular exercise	0.99 (0.81, 1.22)	0.925	1.04 (0.79, 1.36)	0.775
WHR	81.1 (18.7, 351.8)	<0.001	691.1 (112.9, 4228.8)	< 0.001
TC	1.21 (1.10, 1.33)	<0.001	1.29 (1.14, 1.46)	< 0.001
TG	1.12 (1.04, 1.22)	0.004	1.26 (1.16, 1.37)	< 0.001
HDL-C	0.73 (0.54, 0.98)	0.037	0.81 (0.55, 1.20)	0.296
Hypertension	1.78 (1.45, 2.20)	<0.001	2.11 (1.62, 2.75)	< 0.001
Resident (rural)	2.69 (2.18. 3.32)	<0.001	1.38 (1.04, 1.83)	0.024
Diabetes family history	1.52 (1.20, 1.92)	0.001	2.52 (1.88, 3.40)	< 0.001

Multinomial regression model was applied to analyze risk factors of prediabetes and diabetes. Odds ratio (OR) (95% confidence interval) for assessment of associations of risk factors with outcome of prediabetes and diabetes in 3173 study subjects were presented.

## Discussion

The prevalence of diabetes in the present study of Liaoning Province, China was 10.4%, which is close to that of Chinese population 2007–2008 (9.7%) [2] and Korean population 2007–2009 (11.0%) [10]. Overweight and obesity have become a growing global public health problem with increasing prevalence in many countries. Diabetes is considered as complication closely related to obesity [19]. Obesity is usually defined according to WHO criteria which is based on BMI [20], although BMI cannot distinguish between muscle and fat mass. Previous studies have demonstrated that excessive accumulation of adipose tissue in particular in the upper body and abdomen rather than the lower body contributed to metabolic complications such as diabetes [21, 22]. WC and WHR have also been reported to be better discriminators of metabolic risk factors than BMI [23]. Here we used ROC curves to evaluate contribution of the above obesity indices to diabetes risk. WHR appeared to be a better one to predict diabetes risk.

The prevalence of DR in China reported by previous epidemiological studies is different due to study design, sampling size, region, and other complexities. According to a study in Shanghai between the year of 2005 and 2006, which included 1300 subjects aged from 42 to 78, the prevalence of DR was 19.9% in the diabetic population and 8% in prediabetic population [24]. In 2006, a screening study of DR in Beijing among 4439 people with the age of over 40 found 235 diabetes patients with the prevalence of DR as 37.1% [25]. The other study in rural area of China (Handan) in 2007 showed that the prevalence of DR was 14.24% [26]. Our study showed that the prevalence of DR in diabetic people was 11.9%. The prevalence of DR-like retinopathy was 1.4% and 0.3% in prediabeteic and non-diabetic subjects respectively. The prevalence of DR and DR-like retinopathy of the present study was lower than that reported in other domestic epidemiological studies. This difference may be caused by the diversities of population characteristics such as age range, geographic region civilization of resident area and diagnostic criteria of diabetes. The subjects of previous studies were over the age of 30 or 40 [26], diagnosed with diabetes only by fasting blood sugar test [26]. However, in our study, OGTT test was used to confirm the diagnosis of diabetes. Meanwhile we adopted the diabetes diagnostic criteria of ADA [16], which included the diabetic patients whose fasting blood sugar was normal but 2-hr OGTT was up to or higher than 11.0 mmol/L. These patients were 35.40% of all diabetes patients in the present study. Our study used the same method with the epidemiological screening conducted among the population with the age of over 30 in THENI Province of South India from 2005 to 2006 [27], and the results were similar. In addition, 64.90% of the diabetic patients were newly on-site diagnosed in the present study. Their relatively better pysical conditions and shorter disease history may contribute to the low prevalence of DR as well.

Our study shows that duration of diabetes is the main risk factor for DR. Epidemiological data from various studies have identified high blood sugar and hypertension as risk factors for DR [28–30]. We did not see significant association of DR risk with hypertension or blood sugar levels. The ADVANCE study containing 11,140 type II diabetic patients showed that strict management of glycosylated hemoglobin had no effect on the control of 5-year incidence rate of DR [31]. It also indicated that blood pressure control within the normal range (below 140/80 mmHg) had no effect on preventing DR progression [31]. Thus further study on risk factors of DR besides blood sugar and blood pressure is in need.

We also observed that the prevalence of diabetes (9.5%) and DR (10.3% in diabetic patients) in urban areas was significantly lower than that (11.2% and 13.0%, respectively) in rural areas. Due to the development of society and economy in China, people living in some rural areas (as in our study) are experiencing the life-style transformation from agriculture to industry. Their incomes increase, leading to the changes of dietary structure. The etiological factors of diabetes living in the urban and rural areas are not of big difference in this respect. However, the proportion of newly diagnosed diabetic and DR patients in urban areas were significantly lower than that in rural areas. The causes of this regional difference are probably related to the poor recognition of disease prevention, unawareness of the diabetes and lack of treatment due to relatively limited medical resources in rural areas. A study using a linear regression model to examine the association between normalized average HbA1c of 1766 diabetic children and the median household income of their neighborhoods (obtained from Statistics Canada, 2006 Census data) found a negative linear association between the level of income and metabolic control assessed by HbA1c, for every increase of 15000 dollars in annual income, HbA1c decreased by 0.1% [28]. The rural prevalence of DR reported in the present study is lower than that in the Handan of Hebei Province [25], which may be related to different diagnostic criteria and age of study subjects. In the previous study, diabetes was defined as fasting blood sugar levels ≥ 7.0 mmol/l among subjects older than the age of 30. We conducted our study in a population aged from 20 and adopted the diabetes diagnostic criteria of ADA [16] (FPG ≥ 7.0mmol/L or 2-hr OGTT ≥ 11.1mmol/L). In addition, our study subjects were stratified according to the age distribution of 2006 census in China, which may provide a more accurate estimation on regional prevalence of diabetes and DR.

There is much evidence that smoking increases the risk of diabetes [32–35]. But in our study, we are surprised to see that smoking appeared to be a protecting factor against diabetes. Secondary cigarette smoking was reported to be associated with diabetes risk. Sometimes this association can be strong [35]. In the present study, we collected and analyzed data on past and current smoking. Thus, there could be a lot of secondary cigarette smokers in control group, which may diminish the difference between real never-smokers and primary cigarette smokers. Though exercise is generally considered to reduce diabetes risk, the benefit from exercise against diabetes in some studies was not significant [33]. This can be caused by the limitation of sampling size, and other unknown confounding factors. In the present study, exercise was defined as participation in at least 30 minutes of moderate-intensity aerobic activity for no less than 3 days per week. However, the benefit from different types of exercise (e.g. walking, yoga VS basketball) may vary. Especially in Asian countries like China, physical activity of regular people is not as intense as in western countries. Further studies with large sampling size and more detailed analysis of diabetes and exercise types are needed. Hypothyroidism was suggested to be related with islet function and diabetic complications. A cross-sectional study among 1581 people with normal thyroid function shows that TSH has positive correlation with insulin resistance index [36]. Previous study in Beijing Tongren Hospital on 1170 type II diabetes patients finds that the prevalence of visual acuity-threaten DR is much higher in type II diabetic patients combined with subclinical hypothyroidism than those with normal thyroid function [37]. We did not find significant association between DR and TSH level or subclinical hypothyroidism. The present study was a population-based one leading to relatively smaller diabetes and DR population than those hospital-based ones. In our study, the gender composition was different from the general population, which may contribute to bias in the estimation of diabetes prevalence. However, gender-adjust was used during comparison and logistic regression analysis, which should eliminate the bias between different groups.

The total population of Liaoning province was 40,900,000, which accounted for 3.4% of the total population in the mainland of China. The urban population was 21,300,000, which accounted for 52.13% of the total population in the province. It is estimated that there could be 4,333,000 diabetic patients and nearly 610,000 DR patients in Liaoning province according to our study. A great number of diabetic patients may be under risk of DR because of no self-awareness. Therefore, there is a great importance of accelerating the public health education including the prevention of diabetes, as well as establishing a good screening, treatment and follow-up mode of diabetes and DR in the country, especially in rural areas.
